# The Role of Proteases in the Virulence of Plant Pathogenic Bacteria

**DOI:** 10.3390/ijms20030672

**Published:** 2019-02-04

**Authors:** Donata Figaj, Patrycja Ambroziak, Tomasz Przepiora, Joanna Skorko-Glonek

**Affiliations:** Department of General and Medical Biochemistry, Faculty of Biology, University of Gdansk, Wita Stwosza 59, 80-308 Gdansk, Poland; donata.figaj@biol.ug.edu.pl (D.F.); ambroziak.patrycja@gmail.com (P.A.); tomasz.przepiora@phdstud.ug.edu.pl (T.P.)

**Keywords:** plant pathogenic bacteria, bacterial virulence, regulatory proteolysis, extracellular proteases, effector proteases, T3SS

## Abstract

A pathogenic lifestyle is inextricably linked with the constant necessity of facing various challenges exerted by the external environment (both within and outside the host). To successfully colonize the host and establish infection, pathogens have evolved sophisticated systems to combat the host defense mechanisms and also to be able to withstand adverse environmental conditions. Proteases, as crucial components of these systems, are involved in a variety of processes associated with infection. In phytopathogenic bacteria, they play important regulatory roles and modulate the expression and functioning of various virulence factors. Secretory proteases directly help avoid recognition by the plant immune systems, and contribute to the deactivation of the defense response pathways. Finally, proteases are important components of protein quality control systems, and thus enable maintaining homeostasis in stressed bacterial cells. In this review, we discuss the known protease functions and protease-regulated signaling processes associated with virulence of plant pathogenic bacteria.

## 1. Introduction

A typical infection cycle of a bacterial pathogen consists of several stages, as follows: finding and entering a suitable host; dissemination in the host organism; development of disease symptoms; and finally, transmission to a new host. Successful completion of each stage requires the coordinated action of numerous factors produced by the pathogen, termed virulence factors. These molecules are directly responsible for the colonization of the host, evading host’s defenses, and producing disease symptoms. In particular, they facilitate adhesion to the host surfaces; disrupt physical barriers, such as cell walls or intercellular junctions; mediate movement of the pathogen; modulate host’s immune response; and many others. Every stage of the pathogen’s biological cycle is also associated with exposure to a variety of biotic and abiotic, potentially stressful factors that may exert negative effects on the cell. Therefore, microorganisms have developed sophisticated stress response systems that allow for survival under adverse environmental conditions [1,2].

Proteolysis plays various important roles in the mechanisms of virulence over the whole infection cycle of the pathogen. Secreted proteases facilitate penetration and efficient dissemination within the host by participation in the degradation of the host’s physical barriers [3]. Moreover, proteolytic enzymes enable the colonization of the host by combating the host’s defense mechanisms. Several proteases play regulatory roles that allow for the proper response of the pathogen to the environmental changes, and trigger infection at the most appropriate time for the bacteria. Finally, proteases are important components of the protein quality control system that is responsible for maintaining cellular proteostasis. The exposure of a cell to stressful conditions leads to, among others, protein misfolding and denaturation. The irreversibly damaged proteins are removed from the cell by proteolysis so as to avoid their aggregation or malfunctioning [4,5].

The majority of protease functions mentioned above were demonstrated for animal and human pathogens. The involvement of proteases in the virulence of plant pathogenic bacteria has been examined and discussed to a much lower extent. In this review, we present a current state of knowledge on the latter subject. We will focus predominantly on those proteases whose importance for virulence of the most important plant pathogens was well documented. A list of the top 10 bacterial plant pathogens was proposed, as follows: (1) *Pseudomonas syringae* pathovars, (2) *Ralstonia solanacearum*, (3) *Agrobacterium tumefaciens*, (4) *Xanthomonas oryzae* pv. *Oryzae*, (5) *Xanthomonas campestris* pathovars, (6) *Xanthomonas axonopodis* pathovars, (7) *Erwinia amylovora*, (8) *Xylella fastidiosa*, (9) *Dickeya* (*dadantii* and *solani*), and (10) *Pectobacterium carotovorum* (and *Pectobacterium atrosepticum*). All of the listed pathogens are Gram negative bacteria and cause disease in many economically important crops around the world [6]. Their most important properties are summarized in Table 1.

## 2. General Characterization of the Selected Proteases

According to the MEROPS Protease Database [17,18], proteases are divided into the following seven classes: serine, cysteine, aspartic, asparagine, threonine, glutamate, and metalloproteases—depending on their active site composition and mechanism of catalysis. Serine proteases activate the primary hydroxyl group of the active site serine residue, commonly through the “charge-relay” system of the Ser–His–Asp triad or Ser–Lys dyad. The activated hydroxyl group becomes a nucleophile strong enough to attack the carbonyl carbon of the scissile peptide bond. The resulting covalent acyl-enzyme intermediate is then cleaved by hydrolysis. The cysteine and threonine proteases operate by analogous mechanisms, but the active site nucleophiles are represented by a thiol group of Cys or a hydroxyl group of Thr residue, respectively. Aspartic and metalloproteases activate a water molecule to serve as the nucleophile, and do not form a covalently bound complex with substrates [19].

The most abundant bacterial proteolytic enzymes are metalloproteases, serine proteases, and cysteine proteases [20]. The glutamate and asparagine proteases have not been studied much in bacteria, and their role in virulence of phytopathogens is unknown [17,18]. Below, we present a brief characterization of the chosen representatives of the major classes of proteases, whose role in the virulence of the plant pathogenic bacteria is well established.

### 2.1. Metalloproteases

The best characterized metalloproteases in phytopathogens are secreted Prt proteases from *Pectobacterium carotovorum*, *Dickeya dadantii*, *Dickeya chrysanthemii,* and *Xanthomonas campestris* [21,22,23]. Although most of them were identified in the late 1980s and 1990s, their role in bacterial virulence is still elusive. It is expected that these enzymes participate in the degradation of the plant cell wall components (various Prt proteases) and/or take part in combatting the plant immune defenses (e.g., AprA) [21,24].

The characterization of the selected metalloproteases associated with the plant cell wall degradation is presented in Table 2. The role of an individual metalloprotease in bacterial pathogenesis is frequently difficult to establish, as a single bacterial cell secretes several proteases whose functions probably largely overlap and are mutually substitutable. [25]. It is important to note, that the extent of the plant tissue maceration strongly depends on the total number of bacterial cells used for inoculation. The effects of the *prt* mutations in *X. campestris* were more visible when the plant tissues were infected with a low inocula of bacteria [26].

The alkaline protease AprA from the *Pseudomonas syringae* pv. tomato is a homolog of the AprA alkaline protease from *P. aeruginosa*, a pathogen infecting plants as well as animals and humans. AprA is a zinc-dependent metalloprotease from the MA clan, family M10, according to MEROPS [17,18]. The AprA protease is necessary for the full development of *Pseudomonas* virulence in tomato and *Arabidopsis* plant models. The *P. syringae* pv. *tomato aprA* deletion strain is characterized by reduced growth, and fewer disease symptoms were observed in the tomato plants [21].

### 2.2. Cysteine Proteases

#### 2.2.1. YopT Family

The largest group of the effector proteases secreted by the type III secretion system belongs to the YopT (Yop—*Yersinia* outer protein) superfamily of cysteine proteases [34]. These proteins are conserved among the animal and plant pathogenic bacteria, and the model member is the YopY protease from the *Yersinia* genus, a member of the CA clan, C58 family [17,18]. Among the plant pathogens, the AvrPphB (AvrPph3) protein from *P. syringae* pv. *phaseolicola* 1302A is the best characterized representative of this family. The 35 kDa protein is injected into the host cell where it undergoes autocatalytic processing to a mature 28 kDa protease. This cleavage generates a new N-terminal signal for fatty acylation, a modification that is necessary for activity and for the proper membrane localization of AvrPphB [34]. Other thus far identified AvrPphB homologs also localize to the membranes in the host cells, however not all of them undergo autocatalytic processing and lipidation. These include HopN1 (HopPtoN) and HopC1 (HopPtoC) from *P. syringae* pv. *tomato* DC3000, ORF4 from *P. syringae* pv. *phaseolicola* 1449B, and RipT from *Ralstonia solanacerum* GMI1000 [35,36,37].

The crystal structure of the mature AvrPphB protein was solved, and its analysis revealed that the core structure resembles that of the papain-like cysteine proteases. The active site triad consists of Cys98, His212, and Asp227 [38]. The protease was demonstrated to hydrolyze peptide bonds following the Gly–Asp–Lys motif, with the most pronounced role of the residues Gly and Asp in the substrate recognition and binding [38]. Interestingly, the in-silico analysis of the amino acid sequences of the phytopathogen-derived YopT homologs revealed that the predicted substrate binding sites significantly vary among these enzymes. Therefore, a different substrate specificity and, consequently, different cellular targets, are expected [38]. Indeed, the roles and targets described for the various YopT homologs are different, as described in Section 3.2.

#### 2.2.2. SUMO Proteases

To this group belongs the XopD (Xop: *Xanthomonas* outer protein) protease from *Xanthomonas campestris* pv. *vesicatoria* [39]. It is a member of the CE clan of cysteine proteases, family C48 of the SUMO (small ubiquitin-like modifier) deconjugating enzymes [17,18]. XopD is a large (760 amino acids) modular protein composed of the N-terminal DNA-binding domain, which determines the host range; two internal ERF(ethylene response factor)-associated amphiphilic repression (EAR) motifs (^Leu^/_Phe_AspLeuAsn^Leu^/_Phe_(Xaa-any amino acid)Pro); and finally, a C-terminal SUMO protease domain [40]. The EAR motifs occur in the plant repressors that regulate stress-induced genes [41]. The genes encoding the XopD-like homologs were only identified in the bacteria from *Xanthomonas, Acidovorax,* and *Pseudomonas* spp [40,42,43]. The C-terminal domains of the all of the analyzed orthologs contain the conserved catalytic residues (His, Asp, and Cys) found in the SUMO proteases in the C48 family (MEROPS Database). The C-domain exhibits the isopeptidase activity, and is responsible for the removal of the SUMO moiety from the conjugated target proteins. XopD is an enzyme specific to plant SUMOs and it cuts off SUMO after the sequence Met–Leu–His–X–X–Gly–Gly (where X stands for any amino acid). Moreover, the residues at positions 29 and 35 upstream the cleaved bond in SUMO also contribute to the substrate specificity of XopD. The crystal structure of the catalytic core of XopD has been solved, and this domain shares structural features with the yeast Ulp1 protein, the founding member of the Ubiquitin-like protein proteases (ULPs) family [44]. XopD is localized to the nucleus of the plant cell, however other XopD-like proteins may be targeted to different subcellular sites [40].

The proteins that are post-translationally modified by SUMO are involved in many important biological processes (e.g., nuclear transport, cell cycle, transcription, and the stress response) [42]. Sumoylation is reversible, and the de-sumoylation of various cellular factors is a common regulatory mechanism to modulate cell physiology [42]. Hence, *Xanthomonas* secrete XopD to interfere with the host signaling so as to achieve their own benefits.

### 2.3. Serine Proteases

#### 2.3.1. ClpP

The ClpP protease is a member of the clan SK of serine proteases, family S14, according to the MEROPS database [17,18]. The ClpP homologs are endopeptidases characterized by a unique arrangement of the active site triad, Ser–His–Asp. The ClpP monomer is composed of the following three domains: the N-terminal, the “handle”, and the “head”. The functional unit is represented by a large heterooligomer composed of the ClpP protease and Clp unfoldase (ATPase) subunits. There are several Clp unfoldases that associate with ClpP to form active proteolytic machinery, namely: ClpA and ClpX in the Gram negative bacteria, and ClpC, ClpE, and ClpX in the Gram positive bacteria [45]. The unfoldases are ATP-binding proteins that belong to the AAA+ family of ATPases (ATPases associated with diverse cellular activities) [46].

**Figure 1:**
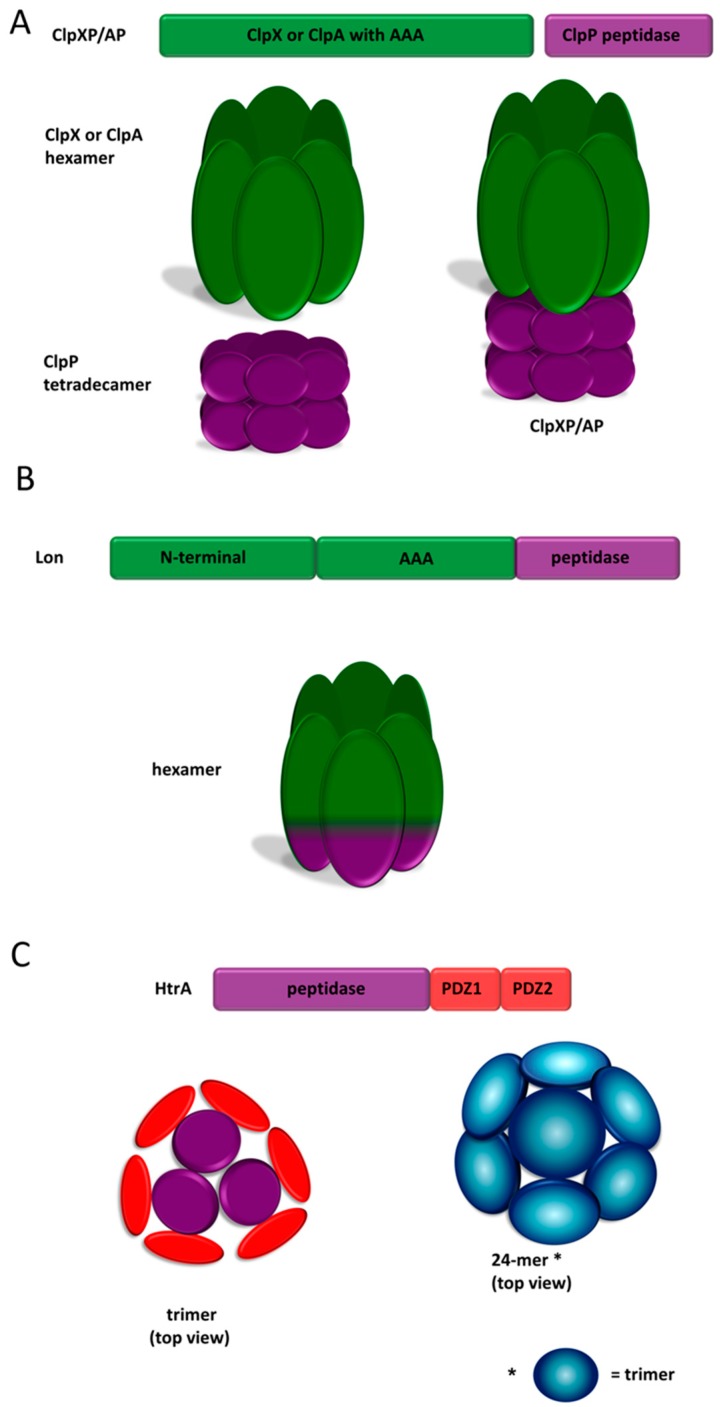
Structures of proteases ClpAP/ClpXP (**A**), Lon (**B**), and HtrA (**C**). Schematic domain structures and exemplary quaternary structures of proteases are presented. The peptidase domain/subunit is shown in purple, the unfoldase domain is green, and the PDZ (post synaptic density protein 95, Drosophila disc large tumor suppressor, and Zonula occludens-1 protein) domain is red.

The ClpXP and ClpAP complexes from *Escherichia coli*, a model Gram negative bacterium, are the best characterized examples of the bacterial Clp proteases [47,48]. ClpP is considered as a self-compartmentalizing protease, as it spontaneously oligomerizes to form a barrel-like structure, formed of two heptameric rings stacked “face-to-face”. The heptamers are formed from the packing of the head domains, while the inter-heptamer connections are mediated by the interactions of the handles. The protease active sites are exposed to the interior of the chamber, and are accessible only via narrow apical and distal pores formed by the N-terminal domains [45] (Figure 1A). The ClpP protein alone can degrade only short polypeptides, and it virtually lacks substrate specificity. Only the interaction with the ClpA or ClpX unfoldase subunits ensures the efficient and specific digestion of the protein substrates. The ClpP tetradecamer associates with one or two hexameric rings of the Clp unfoldase at its apical and/or bottom surface [49]. The unfoldases play three major roles, namely: (1) recognition of proper substrates to be degraded by ClpP, (2) unfolding of the substrate using energy provided by hydrolysis of ATP, and (3) delivery of the unfolded polypeptide into the proteolytic chamber by threading it via the entrance pore [49]. ClpA and ClpX generally show a different substrate specificity, although their preferences overlap to some extent. For example, they both bind the proteins that are C-terminally tagged with the SsrA (tag encoded by small stable RNA A) peptide [50,51]. The tag consists of 11 nonpolar residues [52], and is added to a nascent polypeptide chain whose translation is stalled on the ribosome [50,53]. The specificity of the Clp proteases is further regulated by interactions with the dedicated adaptor proteins. For example, SspB (stringent starvation protein B) translocates the proteins tagged with a SsrA peptide to the ClpXP protease [51,54], whereas ClpS is an adaptor protein responsible for binding to the substrates marked with the N-degron and their delivery to the ClpAP protease [55,56,57]. In the latter case, a protein destined for degradation contains a bulky hydrophobic residue at its N-terminus, or is post-translationally modified at the N-end to introduce a Leu residue [58].

Apart from the housekeeping roles, Clp proteases are involved in regulatory proteolysis. For example, ClpXP degrades the RpoS sigma factor (sigma S) [59], UmuD’ (umu: UV mutagenesis), involved in the error-prone DNA replication [60], a cytoplasmic domain of the RseA anti-sigma factor [61]. ClpAP is known to degrade the Dps (DNA-binding protein of starved cells) nucleoid-condensing protein during starvation stress in the N-end rule pathway [62].

#### 2.3.2. Lon

According to the MEROPS database, the Lon protease belongs to the clan SJ of serine proteases, family S16 (Lon protease family) [17,18]. The active sites of these proteases contain the Ser–Lys dyad. The Lon family members are cytosolic ATP-dependent proteases that are highly conserved among prokaryotes [63]. The Lon homologs from the bacterial phytopathogens are not fully characterized in terms of their structure and activity. Nonetheless, an analysis of their amino acid sequences indicates that all domains of the protein are preserved, and the phytopathogen derived Lon homologs are expected to be structurally and functionally similar to the well described Lon proteins. For example, the *P. syringae* Lon protease seems to be a functional equivalent of its homolog in the bacterium *E. coli* [64]. The Lon (La) protein from *E. coli* is composed of the following three domains [65]: (1) the N-terminal domain responsible for substrate recognition and oligomerization [66,67]; (2) the AAA+ module with Walker A and Walker B ATP-binding motifs [68], responsible for substrate unfolding and translocation to the proteolytic chamber in the ATP dependent manner; and (3) the C-terminal peptidase domain with a Lys–Ser dyad [69,70]. In a solution, Lon forms homooligomeric structures (Figure 1B). In a physiological concentration, the Lon molecules are in a state of equilibrium between the hexamers and dodecamers that are formed by interaction between the N-domains of two stacked hexamer rings [66,71,72]. The dodecamers are characterized by reduced ATPase activity and they poorly degrade the oligomeric substrates in comparison to the hexameric forms [72]. The crystal structure of the entire Lon molecule is not available, however, electron microscopy provided information about the quaternary structure of this protein. The Lon hexamer forms a degradation chamber with active sites exposed to the center, accessible via an axial pore in the AAA+ ring [70,71,72].

Lon is involved in the “housekeeping” protein turnover as well as in regulatory proteolysis. In the first case, it degrades the aberrant protein accumulated in the cell under stressful conditions by recognizing the hydrophobic regions, normally buried in the native protein structures [73]. The protease cuts substrates preferentially after nonpolar amino acids [74], and produces short peptides consisting of 10–20 amino acids [75]. In the case of regulatory proteolysis, the Lon substrates are in their native conformation and the recognition signals for Lon are localized at the N- or C-terminus of the protein substrate [76,77]. However, the precise mechanism of recognition is poorly understood. The proteolytic activity of Lon can be stimulated by protein substrates [78,79,80], inorganic polyphosphates [81], DNA [82] and the hydrolysis of ATP [79]. Known inhibitors of proteolytic activity are, among others, ADP [83,84] and diisopropylfluorophosphate (DFP) [84].

*E. coli* Lon participates in a variety of cellular processes through the degradation of regulatory proteins. It takes part in cell division by degrading the cell division inhibitor SulA [85]. Lon controls capsule production by degrading RcsA, an activator of colanic acid capsular polysaccharide synthesis [86]. Lon protects the cell from error-prone DNA replication in SOS mutagenesis by degradation of UmuD, whose high concentrations indirectly leads to aberrant mutagenesis [77]. Lon participates in the silencing of the response to oxidative and antibiotic stress by degrading SoxS (Sox—Sry-related high-mobility-group box) and MarA (multiple antibiotic resistance activator) transcriptional factors, respectively [87]. It is involved in sulfur assimilation and the biosynthesis of cysteine and methionine by degrading the CysB and MetR transcriptional activators, respectively [88].

In phytopathogens, the Lon homologs also play a variety of functions and are usually required for full virulence. The *P. syringae lon* mutants produce fewer symptoms and show weaker growth in the host plants when compared to the wild type (wt) strains [89]. The *A. tumefaciens lon* mutant is highly attenuated for virulence and yields less tumor mass than the wt bacteria [90].

#### 2.3.3. High Temperature Requirement A (HtrA) Proteases

HtrA (high temperature requirement A) proteins constitute a serine protease family, and according to MEROPS, are assigned to family S1, subfamily S1C, sub-clan PA(S) [17,18]. This family is strongly conserved in the evolution group, which is generally involved in protein quality control [91]. Several HtrAs exhibit an additional, chaperone activity. In bacteria, they are generally responsible for maintaining proteostasis in the extracytoplasmic compartment by degradation of misfolded and damaged proteins, and/or preventing their aggregation. HtrAs were found to participate in maturation and export of the extracellular or membrane proteins, including virulence factors. Some HtrAs also play regulatory roles. Bacterial HtrAs are localized extracytoplasmically in the periplasm, attached to the membranes or secreted out of the cell [91].

HtrA (DegP) from *E. coli* is a model bacterial HtrA. The mature polypeptide is composed of three domains, namely: N-terminal proteolytic and two C-terminal PDZ (post synaptic density protein 95, Drosophila disc large tumor suppressor, and Zonula occludens-1 protein domain). The proteolytic domain is of the chymotrypsin type and consists of two beta-barrels with the active site triad, His–Asp–Ser, located at their interface [92]. The PDZ domains are responsible for substrate binding and maintaining the proper quaternary structure [93,94,95]. All of the known HtrAs form oligomers with trimers being the basic structural units. *E. coli* HtrA assembles into various oligomeric forms, depending on the substrate presence. The resting state of the protease is a hexamer with the active centers being in an inactive conformation. The activation of the enzyme is accompanied with large structural changes, including the rearrangement of the oligomer. The hexamer dissociates to trimers, which then organize into large cage-like structures composed of 12 or 24 subunits (Figure 1C) [96,97]. Alternatively, membrane-associated HtrA forms bowl-like oligomers, which are assumed to be in an active state [98]. The protease is activated on the basis of allosteria primarily, by binding of substrates or suitable peptides to the PDZ1 and proteolytic domains [97,99]. The enzyme shows specificity for the unfolded substrates with exposed hydrophobic residues, and preferentially cleaves bonds after or between paired nonpolar amino acids [100,101]. HtrA is a processive protease and it cuts proteins into 9–20 amino acid long oligopeptides [95].

The best characterized HtrA homologs from plant pathogenic bacteria are the MucD proteins, identified in *Pseudomonas* and *Xanthomonas* species [102,103,104].

## 3. Proteases as Direct Virulence Factors

The successful colonization of the plant host is directly associated with the ability of the bacteria to interfere with the host defense responses. Plant immune systems sense the presence of pathogens or their secreted factors, which leads to the induction of two major defense pathways. The first line of defense, known as pattern-triggered immunity (PTI), depends on the recognition of certain highly conserved in evolution molecules, termed pathogen-associated molecular patterns (PAMPs) (e.g., flagellin and EF-Tu (elongation factor thermo unstable)), by membrane-localized pattern recognition receptors (PRRs) [105,106]. One of the most studied PRRs is FLS2 (flagellin sensing 2) from *Arabidopsis* [107]. FLS2 recognizes bacterial flagellin, and upon binding of the conserved flagellin epitope flg22, it associates with the coreceptor BAK1 (brassinosteroid insensitive 1-associated kinase 1). This event starts the phosphorylation cascade; the cytoplasmic domains of FLS2 and BAK1 become phosphorylated. Next, the active complex activates the BIK1 kinase (botrytis-induced kinase 1), which subsequently phosphorylates the downstream signaling components to trigger the PTI response [106,108]. The second type of defense, termed effector-triggered immunity (ETI), involves the recognition of virulence proteins (termed effectors), which are injected by pathogens directly into the host cell cytosol. The effectors are expected to suppress cellular defenses and cause disease symptoms [109,110,111], although the exact mechanism of their action and their cellular targets are still far from full understanding. The appearance of a particular effector is monitored by a dedicated resistance (R) protein (R-protein) that can directly recognize the injected molecule or sense the effects of its presence (alterations in host’s proteins) [105,112,113]. As a result, several immune responses are induced, including cell wall modification (deposition of lignin and callose); the production of reactive oxygen species, nitric oxide, and antimicrobial compounds; the activation of systemic acquired resistance; and hypersensitive response (HR), leading to localized programmed cell death at the site of infection in resistant plants [112,114,115].

There is a constant evolutionary race between the plant host defenses and phytopathogen attack strategies. Plant defense systems are subject to attack by bacterial virulence factors, which force plants to evolve mechanisms that sense and target the effector molecules secreted by pathogens. This, in turn, exerts an evolutionary pressure to develop further effectors that overcome the action of ETI, and allows for the final establishment of infection in susceptible hosts. In parallel, natural selection leads to the development of new R-protein specificities in plants, so that ETI can act again [112].

### 3.1. Extracellular Bacterial Proteases

#### 3.1.1. Protection against the Host’s Extracellular Defense Mechanisms

**Figure 2:**
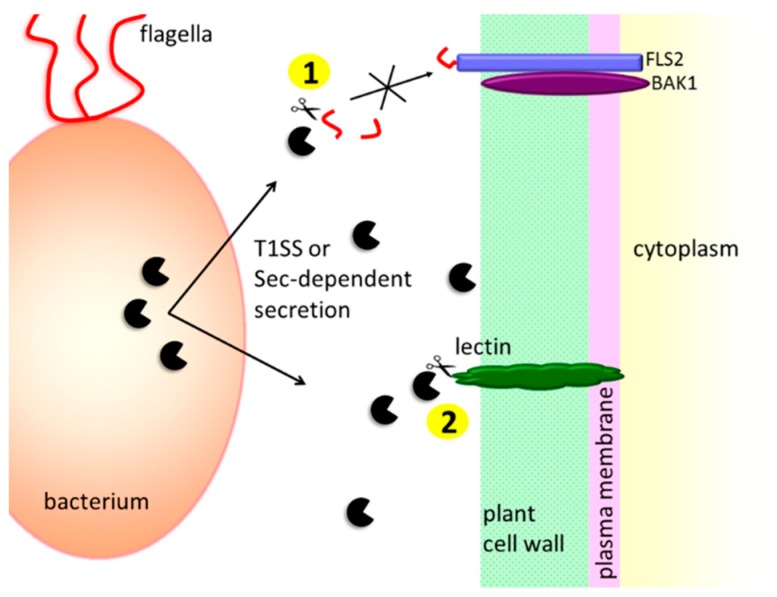
The extracellular bacterial metalloproteases prevent the recognition of the pathogen by the plant, and contribute to plant cell wall degradation. (1) Protease AprA degrades the monomers of flagellin, and thus disables the signaling from the FLS2 receptor [116]. (2) Secreted Prt proteases are believed to participate in the degradation of the plant cell wall. For example, Prt1 from *P. carotovorum* in vitro cuts the plant cell wall structural proteins, potato lectin, and extensins [24].

One of the first events associated with bacterial infection and plant cell immune response is the recognition of PAMPs by the plant cell membrane PRRs to subsequently induce a PTI response. Therefore, bacteria have developed several tactics to avoid being recognized by the host. For example, *P. syringae* pv. *tomato* secretes the AprA protease into the plant apoplast. AprA degrades monomers of flagellin, strong inducers of innate immune responses [21,116]. In the process of assembly of bacterial flagellum, a certain fraction of the flagellin monomers “leaks” into the medium; eventually, flagellin monomer arises from the damaged flagellar filaments [117]. AprA is specific for the flagellin monomers, and it does not cleave the polymeric flagellin. Hence, it prevents flagellin-mediated immune responses without impairing the bacterial motility [116] (Figure 2).

#### 3.1.2. Contribution to the Plant Cell Wall Degradation

Plant pathogenic bacteria enter their hosts using wounds or natural openings (e.g., stomata). Then, depending on the infection strategy, they spread in the plant, sometimes causing systemic infections. Pectinolytic bacteria first localize to the intercellular space, termed apoplast, where they multiply without causing infection symptoms. When a proper cell density is reached, bacteria start producing a set of hydrolytic enzymes, comprising cellulases, pectinases, and proteases [118,119].

As mentioned in Section 2.1, it is presumed that the secreted proteases are directly involved in the degradation of the plant cell wall components, although this function has not been clearly demonstrated in most cases. In vitro, *P. carotovorum* Prt1 was able to degrade potato lectin and extensins isolated from various plants. Both substrates are plant cell wall structural proteins that are expected to stabilize the cell wall structure. Hence, it is possible that the Prt protease supports pectinases and cellulases in destroying the plant cell wall [24] (Figure 2). Certain secreted metalloproteases were also shown to promote cell wall degradation indirectly by processing pectinases. PrtA and PrtC proteases from *D. dadantii* 3937 remove the N-terminal propeptide from PelI-2 (pel—pectate lyase), generating a more active PelI-3 variant. PelI-3 has a higher capacity of potato tuber maceration compared with the PelI-2 pectinase. Moreover, contrary to the unprocessed polypeptide, PelI-3 induced necrosis in the tobaco leaf model [22]. 

### 3.2. Effector Proteins Injected into the Host

Proteases injected into the host cells are important players in bacterial infection strategy. The secretion of the effector molecules, including proteases, is accomplished by the type three secretion system (T3SS), which is considered as one of the major virulence factors in the Gram negative bacteria. T3SS is a syringe-like structure projecting across the bacterial envelope, and the host cell wall and plasma membrane, to reach the host cell cytoplasm [120]. Several reports point to the importance of the T3SS at the initial infection stages in the plant hosts, caused by phytopathogens such as *P. syringae, R. solanacerum,* several *Xanthomonas*, *Erwinia*, and *Pantoea* spp. [121,122,123,124]. In these pathogens, T3SS is referred to as the hypersensitive response and pathogenicity (Hrp) system, and is encoded by *hrp* (hypersensitive response (HR) and pathogenicity) and *hrc* (HR and conserved) gene clusters [112,125,126]. It is necessary for the successful infection of the susceptible host plants, but is also responsible for defense associated HR, rapid localized cell death in the infected plant tissue to restrict pathogen growth [124]. The exact mechanisms of HR are yet to be determined, but it is believed that HR progression is dependent on the direct or indirect sensing of the bacterial effector protein presence by a cognate R protein. The number and types of effectors vary, depending on the bacterial species and strain. For example, the *P. syringae* strains have a repertoire of over 60 effectors in total [109,127].

**Figure 3:**
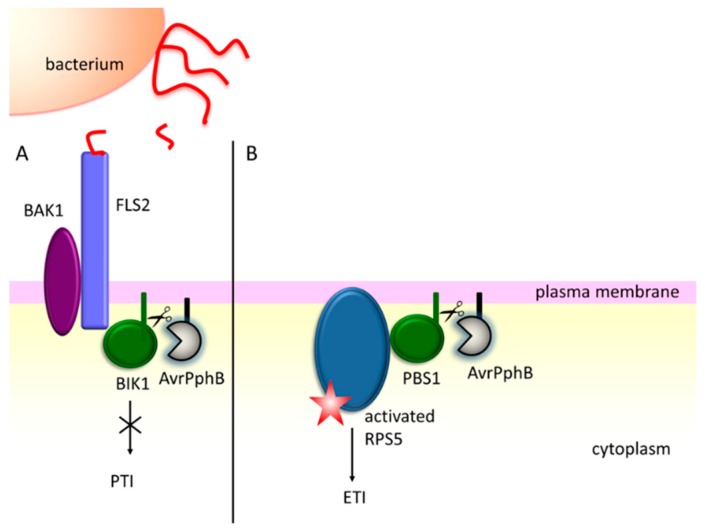
The AvrPphB effector protease from *P. syringae* acts as a double sword in the plant response to bacterial infection. (**A**) After the recognition of flagellin by FLS2, AvrPphB degrades the botrytis-induced kinase 1 (BIK1) of the active receptor complex and blocks the pattern-triggered immunity (PTI) pathway [130,131]. (**B**) Alternatively, AvrPphB may cut the PBS1 protein, which is guarded by the RPS5 (resistance to Pseudomonas syringae 5) protein. RPS5 becomes activated and induces the effector-triggered immunity (ETI) response [134].

Secreted effector proteins frequently manipulate host signaling pathways [128]. For example, certain effector proteases cleave kinases and thus affect phosphorylation cascades. To this group belongs the AvrPphB protease from *P. syringae* pv. *phaseolicola*. The transgenic *AvrPphB Arabidopsis rps5-2* susceptible mutant showed a reduced PAMP response, including the lower deposition of callose, and decreased the accumulation of H_2_O_2_ [129]. AvrPphB cuts the PBS1(AvrPphB susceptible1)-like kinases of the receptor-like cytoplasmic kinase (RLCK) subfamily VII, including BIK1 and PBL1 (PBS-Like 1) kinases, whose activity is required for signaling from multiple PAMPS (e.g., flagellin or chitin) and the induction of PTI defenses [130,131]. BIK1 associates with the PRR complex to regulate immune responses by phosphorylating downstream components [129,132,133], hence, the cleavage of this kinase supresses the PTI signalling (Figure 3A).

AvrPphB cuts also the PBS1 (AvrPphB-susceptible) protein kinase. However, the role of PBS1 in plant defense mechanisms is different from those played by BIK1 and PBL1, as the *pbs1* Arabidopsis mutants show only minimal defects in PTI [129]. PBS1 is required for HR in the resistant plants, and is guarded by the R protein, RPS5 (resistance to Pseudomonas syringae 5) [134]. According to the “guard” hypothesis, PBS1 is regarded as a bait, in order to detect the specific effector protein [105]. When AvrPphB cleaves PBS1, RPS5 becomes activated and induces ETI [134] (Figure 3B).

**Figure 4:**
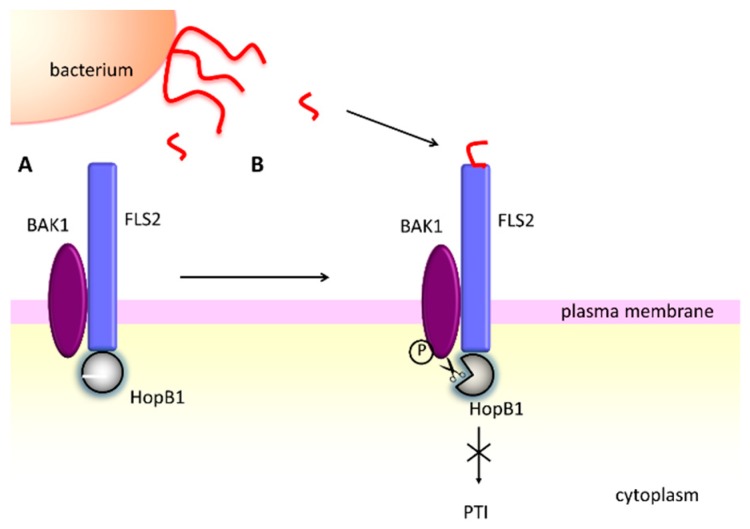
The HopB1 effector protease from *P. syringae* suppresses the PTI plant response [135]. (**A**) HopB1 constitutively interacts with the unstimulated FLS2 receptor. (**B**) When the activated receptor complex is formed, HopB1 degrades phosphorylated BAK1 and blocks the PTI response.

As demonstrated above, AvrPphB acts as a double-edged sword, promoting bacterial infection, but also disclosing the presence of the pathogen in the hosts equipped with the specific R-protein. A recently identified effector protease, HopB1 from *P. syringae* pv. *tomato,* allows for blocking the PTI response without evoking the ETI resistance [135]. HopB1 is a T8 family threonine protease (MEROPS accession number T08.001) that specifically cuts certain SERK (somatic embryogenesis receptor like kinase) kinases, including the cell-surface immune co-receptor BAK1. HopB1 is constitutively bound to FLS2. The protease hydrolyzes the peptide bond between residues Arg297 and Gly298 in the P loop of the co-receptor BAK1, and the corresponding residues in the homologous SERK kinases. Importantly, cleavage occurs only when BAK1 is activated by the perception of flagellin (Figure 4). Thus, HopB1 does not interfere with the non-activated immune receptors, and this is probably a reason it does not induce the effector triggered immunity [135].

**Figure 5:**
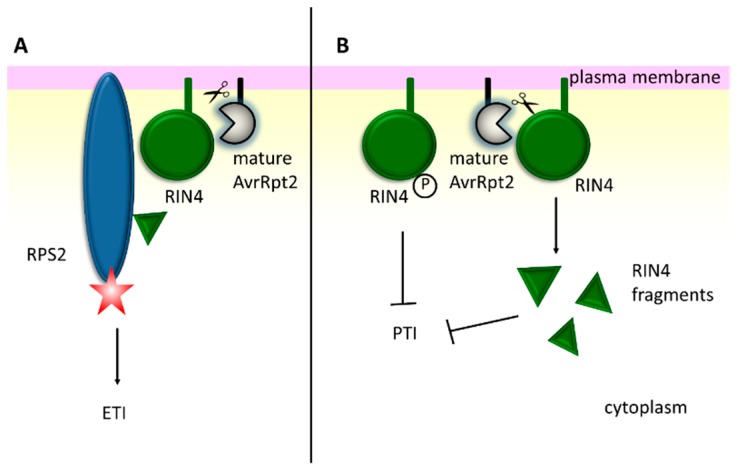
The AvrRpt2 effector protease from *P. syringae* suppresses the PTI plant response, but may induce the ETI signaling. One of the AvrRpt2 targets is a membrane protein RIN4 (RPM1-interacting protein 4). (**A**) In the resistant plant, cleavage of RIN4 is sensed by the RPS2 protein, whose activation triggers the ETI pathway [105]. (**B**) In the sensitive plant lacking RPS2, the degradation of RIN4 by AvrRpt2, or its phosphorylation by other bacterial effectors, generates products that efficiently inhibit PTI signaling [142,143]. Star, arrow and T-bar stand for activated RPS2 protein, stimulation and inhibition, respectively.

AvrRpt2 is a cysteine protease of *P. syringae* pv. *tomato* that undergoes activation and autoprocessing within plant cells [136,137,138]. The activation of AvrRpt2 requires conformational changes induced by the host cell general folding catalyst, cyclophilin (e.g., *Arabidopsis* ROC1). Cyclophilin binds AvrRpt2 and properly folds it by peptidyl-prolyl cis/trans isomerization. The activated AvrRpt2 then cuts its N-terminal 71 amino acids away, and the truncated form localizes to the plasma membrane, where its substrates reside [138,139]. AvrRpt2 induces the HR response in *Arabidopsis* by targeting RIN4 (RPM1(resistance to *Pseudomonas syringae* pv. *maculicola*1)-interacting protein 4), a plasma membrane associated protein [137,140]. RIN4 is a target for multiple effectors, however, its role in plant immunity is not fully understood. Presumably, it is implicated in the regulation of stomatal opening [141]. RIN4 is guarded by two R proteins, RPM1 and RPS2. The first one senses the phosphorylation of RIN4 as a result of effector proteins AvrB and Rpm1, and the other perceives the cleavage of RIN4 by AvrRpt2. Hence, the proteolytic activity of AvrRpt2 produces signals to induce ETI in a resistant plant [105] (Figure 5A). However, in the absence of RPS2, the cleavage of RIN4 suppresses the host’s ability to respond to the presence of AvrB or AvrRpm1. Interestingly, RIN4 exhibits an anti-PTI activity, and the phosphorylated form of RIN4 acts stronger than the non-phosphorylated one [142]; moreover, RIN4 fragments suppress PTI more efficiently than membrane-anchored RIN4 [143]. Hence, targeting RIN4 provides one of the strategies to inhibit pathogen recognition (Figure 5B). The RIN4 protein belongs to the plant-specific nitrate-induced (NOI) family [144]. It was demonstrated that AvrRpt2 was able to cleave almost all of the NOI family members (14 out of 15). The consensus amino acid sequence for cleavage was determined as follows: [LVI]PxFGxW (where x stands for any amino acid) [145]. 

AvrRpt2 is also shown to be involved in the RIN4 independent mechanisms of plant immunity modulation. It disrupts the action of mitogen-activated protein kinase (MPK) cascade; in particular, it prevents MPK4 and MPK11 from flagellin-induced phosphorylation. The way in which AvrRpt2 prevents kinase activation is unknown. The AvrRpt2 protease activity is essential to modulate signaling, but the MPK4/11 protein levels were not changed by AvrRpt2. It is hypothesized that mitogen-activated protein kinase kinases (MKK) may be indirectly targeted by AvrRpt2 [145].

The manipulation of the plant hormone signaling is another strategy employed by phytopathogens to establish infection. Plant hormones play important roles in the host defense responses (“stress” hormones), and they regulate plant growth and development (“growth” hormones). To the first group, the following major hormones belong: salicylic acid (SA), jasmonic acid (JA), and ethylene (ET). The main strategy of phytopathogens is to suppress accumulation and/or block the signals produced by these phytohormones [146], and proteolysis is an important part of this process. The XopJ protease from *X. campestris* pv. *vesicatoria,* a T3SS effector protein from the YopJ cysteine protease family, interferes with the SA signaling by direct targeting of the proteasome. XopJ disrupts the turnover of NPR1 (nonexpressor of pathogenesis-related1), the master regulator of salicylic acid responses. The protease degrades the RPT6 (regulatory particle AAA-ATPaseE6) protein of the 19S proteasome regulatory subunit. This leads to the inhibition of the proteasome and to the accumulation of ubiquitinated proteins, including NPR1 [147,148]. NPR1 must be constitutively removed by the proteasome from the nucleus in order to maintain the SA-responsive gene expression [149]. As a consequence, the SA-dependent defense responses are disrupted and the onset of necrosis in the infected plant tissue is attenuated [147,148]. RPT6 is also a target for the another YopJ family member, HopZ4 (hypersensitivity and pathogenesis-dependent outer protein Z4), from *P. syringae* pv *lachrymans* [150]. 

Jasmonic acid/ethylene pathways act antagonistically to the SA pathway. Therefore, certain phytopathogens take advantage of this fact and activate JA signaling. In an unstimulated plant cell, JA-dependent responses are repressed by a group of proteins termed the jasmonate zimdomain proteins (JAZs). JAZs bind to and repress a series of the JA-responsive transcription factors. The de-repressing of JA signaling occurs as a result of the proteasome-dependent degradation of the JAZs [151]. Bacterial pathogens employ various tactics to promote the degradation of JAZs; one of them is a direct cleavage by an effector protease [146]. The HopX1 cysteine protease from *P. syringae* pv. *tabaci* was shown to destabilize JAZs in *Arabidopsis,* and to induce a JA pathway. As a consequence, the stomata became opened, facilitating the entry of bacteria into the plant host, and the SA dependent defenses were inactivated, promoting bacterial infection [152]. Phytopathogens also manipulate the ET pathway. First of all, ethylene is produced by many bacterial species, for example, *R. solanacerum* and *P. syringae* [153,154], and this ability is directly correlated with *Pseudomonas* virulence [155]. It is speculated that the secretion of ethylene disturbs the balance of the plant defense responses depending on this hormone [154]. The ET pathway may be also manipulated by the T3SS effector protease, XopD, from *Xanthomonas campestris* pv. *vesicatoria*. XopD directly targets the ethylene responsive transcription factor SlERF4 of the tomato host plant, which is involved in ET synthesis. XopD is a SUMO protease and destabilizes SlERF4 by its desumoylation, thus increasing its susceptibility to 26S proteasome-mediated proteolysis [156]. As a result, this leads to a reduced ET accumulation and increased susceptibility of the plant host to *X. vesicatoria*. Moreover, the presence of XopD delays the ET-regulated development of leaf chlorosis and necrosis, which benefits the growth of the pathogen by extending the infection period [43,156].

Auxin is a plant hormone that affects almost all aspects of growth and development [157,158]. It acts as a negative regulator of plant immunity, and therefore auxin signaling is repressed during infection [159,160]. The effector protease AvrRpt2 is associated with the promotion of auxin signaling upon *P. syringae* infection. The expression of AvrRpt2 in protoplasts was found to induce auxin-responsive genes [161]. The protease induces the proteasome dependent degradation of two Aux/IAA transcriptional repressor members, auxin-resistant 2 (AXR2) and AXR3 [162]. However, the precise molecular mechanism of this process remains unknown.

HopN1 (HopPtoN) from *P. syringae* pv. *tomato* DC3000 uses another strategy to prevent the HR response. It is a cysteine protease from the YopJ family with a classical catalytic triad consisting of Cys172, His283, and Asp299 [34]. The presence of this protease in the plant is associated with the suppression of pathogen-induced necrosis in both susceptible and resistant host plants [163]. HopN1 localizes to chloroplasts, where it interacts with the PsbQ protein [164]. PsbQ is a component of the oxygen evolving complex (OEC) of photosystem II (PS II), and is involved in the stabilization of this complex under stress conditions [165,166]. HopN1 was found to degrade PsbQ, which led to a reduction of the photolysis of water, consistently lowering oxygen production and electron transport. Moreover, a lack of PbsQ was connected with the reduction of reactive oxygen species (ROS) production in the plant cells. Hence, it is hypothesized that HopN1 suppresses the HR-related localized cell death by blocking the chloroplast-dependent generation of ROS [164]. 

## 4. Regulatory Proteolysis

Proper cellular response to environmental signals is crucial for successful infection, and it requires efficient functioning of a complex network of regulatory systems. Rapidly changing and potentially harmful conditions are sensed by bacteria, and dedicated cellular stress responses are induced [167,168,169]. Alterations in the gene expression and protein activity may promote the elimination of a stress agent and/or the repair of cell damage, and enable bacteria to become stress resistant. The induction of the infection process is strictly controlled as well [170].

Proteases are key components of regulatory networks as the enzymes responsible for the timely and specific degradation of regulatory proteins in response to specific signals. A good example is the Clp protease, known to regulate the activity of the alternative sigma factors. In *E. coli,* ClpP in complex with the ClpX chaperone modulates the sigma E (RpoE) dependent extracytoplasmic stress response. In particular, it degrades the cytoplasmic domain of the RseA protein, an anti-sigma factor. Sigma E, when liberated from the inhibitory interaction with RseA, binds to the core of RNA polymerase [171]. The other sigma factor, RpoS, a master regulator for the general stress response in several Gram negative bacteria, is also controlled by ClpXP [59]. In the exponentially growing bacteria, the level of RpoS is kept low, mainly because of its rapid degradation by ClpXP [172]. The recognition of RpoS by ClpXP is facilitated by an adaptor protein, RssB [173,174]. Under conditions unfavorable for bacteria (stress and starvation), inhibitors of RssB and the depletion of ATP allow for the stabilization of RpoS [175,176,177] and the subsequent activation of the genes involved in cell survival.

In many Gram negative plant pathogenic bacteria, both stress response systems are present (at least at a gene level, according to the NCBI database [178]. However, only the relationship of ClpXP and RpoS was demonstrated in phytopathogens, whereas the involvement of ClpXP in the regulation of the sigma E dependent stress response still awaits experimental verification [179,180].

**Figure 6:**
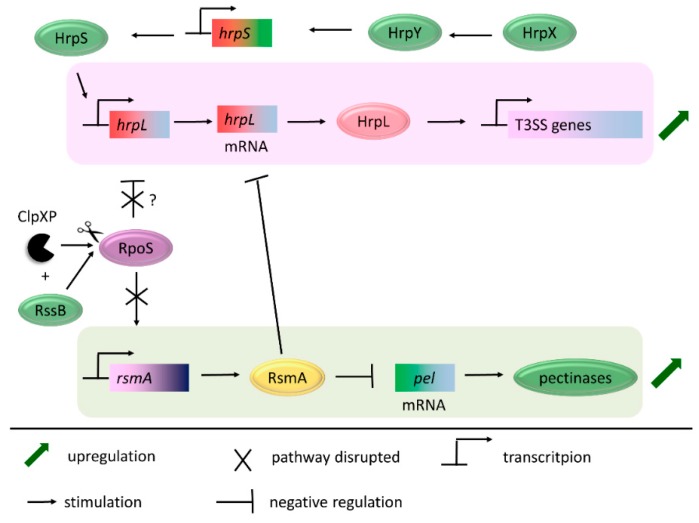
ClpXP positively regulates the expression of virulence associated genes in *D. dadantii*. ClpXP, with the assistance of the adaptor protein RssB, degrades RpoS (sigma S), an alternative sigma factor and global regulator of transcription. RpoS negatively regulates the activity of the *hrpL* promoter (by unknown mechanism), resulting in lower levels of hypersensitive response and pathogenicity (Hrp)-L, which is also an alternative sigma factor and is required for the transcription of the T3SS genes. RpoS stimulates the expression of *rsmA*, whose product destabilizes mRNA molecules, including *hrpL* mRNA and pectinase mRNA. In the absence of RpoS, the synthesis of T3SS and pectinases is stimulated. Figure based on the literature [179].

In *D. dadantii* and *E. amylovora,* the RpoS protein level was shown to be directly limited by the ClpXP proteolytic complex with assistance of RssB. The *clpXP* mutants of both bacterial species accumulate RpoS, and the increased content of RpoS has a direct impact on the expression of several virulence factors. First of all, the T3SS gene expression is downregulated. The aspect of ClpXP-dependent regulation of the T3SS pathway was studied in detail in *D. dadantii* 3937 [179]. As shown in Figure 6, the genes coding for T3SS are under the direct transcriptional control of the HrpL protein, a member of the extracytoplasmic function (ECF) family of alternative sigma factors. The transcription of the *hrpL* gene is regulated by the HrpX/HrpY–HrpS regulatory pathway, while the stability of the *hrpL* mRNA is controlled by the RsmA/*rsmB* sRNA. The functioning of both regulatory systems is RpoS dependent, and, as a consequence, also ClpXP dependent. RpoS was found to negatively regulate the *hrpL* promoter activity (by an unknown mechanism) and also to destabilize *hrpL* mRNA by stimulation of the *rsmA* expression. RsmA (CsrA) (a small RNA-binding protein) is a global regulator of the posttranscriptional gene expression in diverse bacterial species, and its activity is modulated by non-coding regulatory RNA molecules, termed small RNAs (sRNAs) (e.g., *rsmB* RNA (CsrB)). RsmA can bind and destabilize the target mRNA, including *hrpL* mRNA [181].

Accumulation of RpoS affects also the expression of other important virulence related factors. *D. dadantii* mutants lacking *clpXP* or *rssB* (thus accumulating RpoS) were characterized with reduced pectinolytic activity, whereas the *rpoS* mutant showed increased pectate lyases activity, compared to the wt bacteria. In another pectinolytic bacterium, *P. carotovorum,* the production of pectinolytic enzymes was suppressed by RsmA [182]. Therefore, it was speculated that the effect of the *clpXP* or *rpoS* mutation on the Pel production in *D. dadantii* 3937 may be caused by their impact on the *rsmA* gene expression [179]. In the other plant pathogen, *E. amylovora,* elevated levels of RpoS led to a decrease in the production of an important virulence factor, amylovoran. This exopolysaccharide plays important roles in virulence, biofilm formation, and bacterial survival. It was shown that amylovoran was hardly detectable in the *clpXP* mutant bacteria. The opposite effect was observed in the *rpoS* mutant, where the amylovoran production was markedly stimulated as compared with that of the wt *E. amylovora* [180].

**Figure 7:**
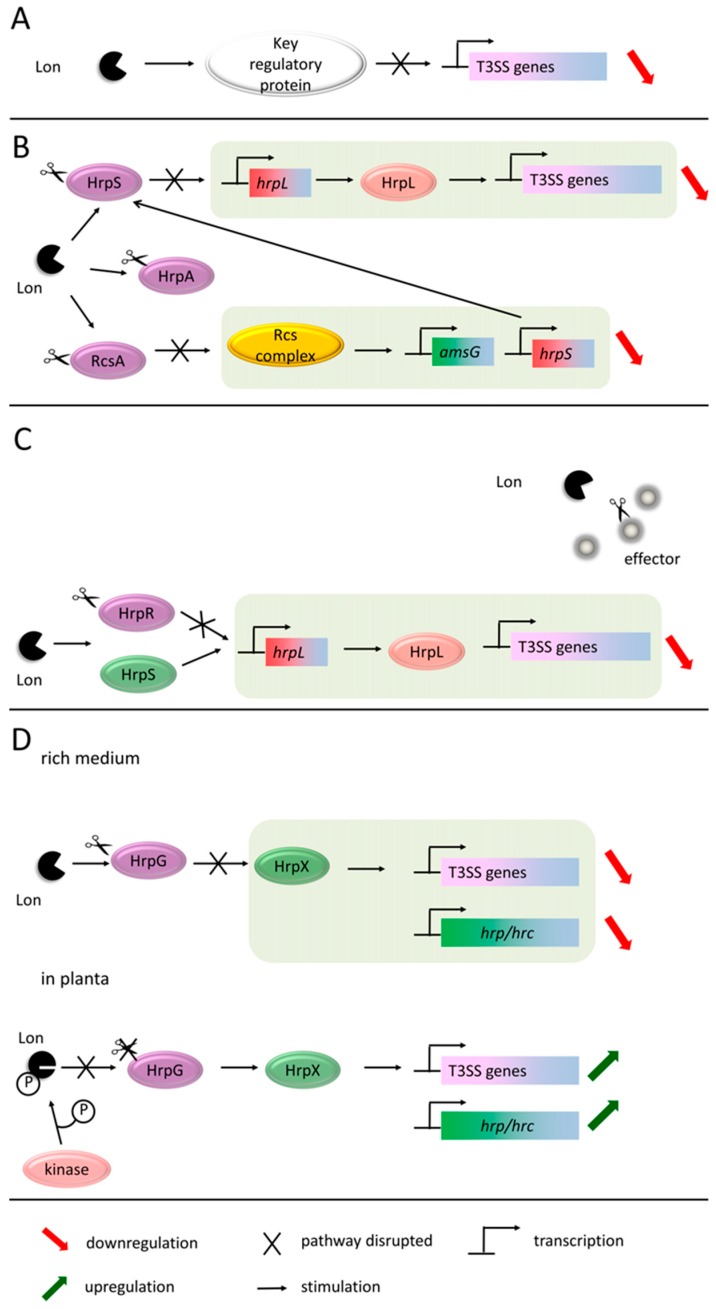
The Lon protease acts as a negative regulator of the Type III secretion system. (**A**) Lon functions through the degradation of the key regulatory proteins—a general scheme [64,180,183]. (**B**) In *E. amylovora,* Lon negatively regulates T3SS at multiple levels. First of all, it degrades HrpS, a positive regulator of the *hrpL* gene expression [180]. Moreover, Lon cuts the RcsA component of the RcsA/RcsB complex, involved in activation of the *hrpS* gene transcription. Lon also targets directly components of T3SS (e.g., it degrades HrpA, a structural protein of the pilus) [184]. (**C**) *P. syringae* Lon downregulates the T3SS expression by the proteolysis of HrpR, a component of the enhancer complex HrpR–HrpS that stimulates transcription from the *hrpL* promoter [64]. Additionally, Lon degrades the effector molecules prior to their export [187]. (**D**) In *X. citri* subsp. *Citri,* phosphorylation on Lon is responsible for the differential expression of T3SS in the external and host environments [183]. Under non-host conditions, Lon is an active protease and it degrades HrpG, a master regulator of T3SS. A deficiency of HrpG prevents the *hrpX* gene expression, whose product is necessary for the transcription of the T3SS associated genes. In planta Lon becomes phosphorylated, which results in the loss of its proteolytic activity. HrpG is stabilized and T3SS is “turned on”.

The second major bacterial cytoplasmic protease, Lon, is also known to regulate virulence traits in several pathogenic bacteria, including plant pathogens. For example, the absence of Lon caused a global change of gene expression in *P. syringae* pv. *tomato* [89]. In particular, Lon acts as a negative regulator of the T3SS expression and synthesis of flagella, and is also involved in cell division and the production of exopolysaccharides (Figure 7A) [64,180,183]. 

The involvement of Lon in the networks controlling the virulence of *E. amylovora* was extensively studied by Lee and colleagues [180]. They showed that the disabling of the *lon* gene resulted in an increased expression of T3SS, and an overproduction of amylovoran and non-motile phenotype. As shown in Figure 7B, Lon degrades HrpS, the key regulatory protein necessary for the transcription of the T3SS genes. Moreover, Lon directly targets a structural protein of the T3SS pilus, HrpA. The half-life of both proteins was markedly increased in the *lon* mutant [184]. The Lon protease is also implicated in the regulation of amylovoran production and motility [184,185]. In these cases, Lon degrades a component of the Rcs (regulator of capsule synthesis) system, RcsA. Rcs is a two-component system including three core proteins, RcsB, RcsC, and RcsD, and one auxiliary protein, RcsA. It was demonstrated that the *lon* mutant cells accumulated a RcsA–RcsB heterodimer. This Rcs complex is known to regulate the expression of several genes by binding to the specific DNA sequence, RseAB box. The expression of *amsG*, the first gene of the amylovoran operon, is stimulated by RcsA/B, whereas the transcription of *flhD*, coding for the master regulator of flagellar biosynthesis, is repressed. Also, the *hrpS* gene is upregulated by the RcsA/B complex, leading to an increased T3SS gene expression [184]. 

In another plant pathogen, *P. syringae,* Lon degrades a regulator of the T3SS expression as well. This bacterium produces two homologous Hrp enhancer binding proteins, HrpR and HrpS, that form an active hexameric complex for the *hrpL* promoter [186]. Lon degrades HrpR (but not HrpS) under non-inducing conditions (e.g., nutritionally rich), and thus represses the expression of the *hrpL* gene [64] (Figure 7C). Once the Hrp pathway is activated, Lon regulates the stability of the T3SS effector proteins prior to secretion. It was demonstrated that in the Lon producing cells, several effectors were unstable, whereas the half-lives of these proteins were increased in the *lon* mutant [187]. As a consequence, the *P. syringae lon* mutants hyper-secreted effector proteins (e.g., AvrPto), which led to the development of rapid plant responses [64]. The other phenotypic features of the *lon* mutant include increased sensitivity to UV, abnormal morphology, and an increased length of cells [64]. This implies the involvement of Lon in the modulation of the other regulatory protein, the SOS-induced cell division inhibitor SulA [188]. The increased level of SulA is sufficient to block septation in *E. coli* [188]. 

The environment-dependent switch to stimulate/inhibit the activity of Lon has long remained a mystery. A recently published work of Zhou and colleagues [183] on the Lon protease from *Xanthomonas citri* subsp. *citri* has shed more light on this subject. The authors found that the activity of Lon may be regulated by means of phosphorylation (Figure 7D). In particular, a single serine residue (Ser 654) within the peptidase domain becomes phosphorylated when bacteria infect the plant host, and this modification inactivates Lon. Conversely, in a rich medium, Lon stays predominantly in a dephosphorylated and active form [183]. The environment-dependent switch of the Lon activity due to the protease phosphorylation nicely explains the mechanism of the T3SS regulation in *Xanthomonas.* One of the Lon substrates in *X. citri* is HrpG, a master regulator of the T3SS system. HrpG stimulates the expression of the transcriptional activator HrpX, which in turn activates the expression of *hrp/hrc* and T3E-encoding genes [189,190,191]. In a rich medium, Lon is active, therefore the cellular level of HrpG is very low; conversely, upon infection of the host plant, phosphorylation inactivates Lon, and HrpG becomes stabilized [183,192]. Consequently, the expression of the T3SS system is suppressed during the non-infectious stage, but induced in the host [193]. 

## 5. Maintenance of Cellular Proteostasis

Most pathogens encounter rapid and often dramatic changes in their external environment during their infection cycle. Entry and establishment in the host are associated with exposure to a variety of unfavorable conditions that may damage cellular components. Upon bacterial infections, plants produce reactive oxygen species, such as hydrogen peroxide and superoxide anions, and this response is termed “oxidative burst” [194]. The pH of apoplast may also be subject to change from being initially acidic, to alkaline [15]. In the non-host environment, soil or groundwater, at the plant proximity, within the phyllosphere and the rhizosphere of plants, phytopathogens are frequently exposed to ultraviolet radiation, low- or high-water availability, diurnal or seasonal temperature changes, fluctuating salinity and/or acidity, and to the presence of antimicrobial plant metabolites [195]. Exposure to stressful conditions damages various cellular components, including proteins, leading to their unfolding or misfolding [196]. In response to the presence of abnormal proteins, a set of proteins, collectively named “protein quality control system”, is induced, whose role is to prevent aggregation and refold polypeptides, or to remove the nonfunctional proteins by proteolysis. The synthesis of several housekeeping proteases is induced under stressful conditions, including Lon, Clp, HtrA, and Tsp homologs [197,198].

The cellular envelope, as the outermost layer, is especially exposed to the external stressful factors. The extracytoplasmic protein quality control system is responsible for maintaining the proteostasis in this compartment. Proteases from the HtrA family and Tsp (tail-specific protease) (also termed Prc) homologs are important components of this system in phytopathogens [102,199]. In *Xanthomonas oryzae* pv. *oryzae,* the Prc protease contributes to multiple cell-envelope stress responses. The *prc* deficient bacteria were susceptible to salt and oxidative stresses, as well as to the presence of sodium dodecyl sulfate (SDS), and consequently, were less virulent to the susceptible plant host *Oryza sativa* cv. IR24 [199]. The *Pseudomonas* MucD protease, an HtrA family member, is also an important component of the envelope quality control system. In *P. aeruginosa,* MucD alleviates periplasmic stress by degrading the misfolded outer membrane proteins [102]. *P. aeruginosa mucD* shows increased sensitivity to thermal and oxidative stresses, and the virulence of the strain is reduced in the *Arabidopsis* model [103]. However, a lack of MucD in *X. campestris* pv. *campestris* does not markedly affect the growth of bacteria under stressful conditions [104]. Hence, the significance of MucD for the survival of bacteria under adverse conditions is not obvious, and seems to be species dependent.

The Lon and Clp proteases are responsible for maintaining homeostasis in the cytoplasm. Their role is especially important under conditions affecting protein folding/stability, therefore, their synthesis is usually upregulated in the stressed cells. For example, the transcription of *lon* and *clpP* is induced in response to salt stress in *D. dadantii* [200], or treatment with acetate of *P. sringae* pv*. phaseolica* [201]. In *A. tumefaciens*, the expression of *lon* was upregulated under heat shock conditions [90]. It is expected that both proteases protect cytoplasm from negative consequences of stressful conditions exerted by the host defense mechanisms, as well as those present out of the host. 

## 6. Conclusions

Intensive research carried out over the last decades has contributed to a significant expansion of knowledge about bacteria–plant interactions. Several bacterial and plant defense mechanisms, as well as pathogen attack strategies, have been revealed. The pathogen–host relationship can be considered as a continuous arms race, and proteases are very powerful weapons. Plants have developed several defense responses, including PTI, ETI, and hormone regulated immunity, and they perceive the presence of phytopathogens by PRR or R proteins. In turn, bacteria have evolved mechanisms that allow them not only to avoid recognition, but also to manipulate the host signaling to achieve their own benefits. It turns out that proteases play key roles in the processes associated with infection. The examples shown in this review underline the role of proteolysis in combatting plant immune responses, as well as in the regulation of virulence traits in bacterial cells. It is no wonder that many protease deficient mutants are attenuated or their virulence is diminished.

Despite the great progress of research in the field of phytopathogen derived proteases, many issues remain unexplored. The mechanisms for regulating most of these proteases are unknown. There is very little information about the structure of the plant bacterial proteases and their specificity, and the available data relate mainly comparative analyses with homologs derived from human and animal pathogens. Hence, extensive biochemical and structural studies on these enzymes are needed in order to fully understand their roles in the virulence of plant pathogenic bacteria.

## Figures and Tables

**Table 1:** Characteristic of selected plant pathogenic bacteria.

Bacterial Pathogen	Plant Host	Disease	Disease Symptoms	References
*Pseudomonas syringae*	Wide range (e.g., tomato, beans, horse-chestnut, and tobacco), depending on the pathovar.	Bacterial speck, halo blight, and bleeding canker	Chlorosis, necrosis, cankers, blights, and water-soaked lesions	[7]
*Ralstonia solanacearum*	Two hundred plant species (e.g., potato, tomato, tobacco, eggplant, ornamentals, and banana)	Brown rot, bacterial wilt, and Moko disease of banana	Plant wilting and rotting	[6,8]
*Agrobacterium tumefaciens*	Wide range (e.g., woody ornamental shrubs, vines, shade trees, fruit trees, cherry, berry, walnut, and herbaceous perennials)	Crown gall tumor	Neoplastic and in consequence limiting plant’s growth	[6,9]
*Xanthomonas oryzae* pv*. oryzae*	Rice	Leaf blight and leaf streak	Pale-green to grey-green and water-soaked streaks near the leaf tip and margins	[6,10]
*Xanthomonas campestris*	A large number of species of the *Brassicaceae* family, such as pepper, tomato, and cotton	Black rot	Blackening of the leaf veins	[6,11]
*Xanthomonas axonopodis*	Wide range (e.g., citrus, cassava, mango, ornamentals, and bean)	Bacterial blight and citrus canker	Angular leaf spots and leaf wilting	[6,12]
*Erwinia amylovora*	young fruit trees (apple, pear, quince, blackberry, and raspberry), and rosaceous ornamentals	Fire blight	Grey-green water soaking and necrosis	[6]
*Xylella fastidiosa*	Wide range (e.g., citrus, peach, elm, oak, oleander, maple, sycamore, coffee, peach, mulberry, plum, periwinkle, and pear)	Pierce’s disease and leaf scorch disease	Chlorosis and premature abscission of leaves and fruits	[6,13,14]
*Dickeya dadantii* and *solani*	Wide range (e.g., potato, rice, maize, pineapple, banana, and chicory)	Blackleg and soft rot	Stem and tuber rotting	[6,15]
*Pectobacterium carotovorum*	Wide range (e.g., potato, ornamentals, cabbage, and carrot)	Soft rot	Tuber, stem, and leaves rotting	[6,16]

**Table 2:** Characteristic of selected extracellular proteases.

Protease Name	MEROPS	Co-factor	Inhibitors	Processing	Secretion Conditions	Gene Expression	Role in Virulence	Proteolytic Activity	Additional Features	References
Prt1 from *P. carotovorum* subsp. *carotovorum* EC14	Clan MA, Family M4	Zn^2+^ not Ca^2+^	Phenan-throline, phosphoramidon, EGTA, Fe^2+^, and Cu^2+^	N-terminal pre-pro-processing	Not secreted in the rich medium; induction of secretion by gelatin	Induced in planta	Unknown	Optimal temperature is 50 °C, optimal pH is 6.0; activity against gelatin casein, potato lectin, ribonuclease A, and BSA; peptide bond cleavage preferentially after proline followed by Ala, Val, or Phe	Low thermal stabiity	[24,27]
PrtW from *P. carotovorum* subsp. *carotovorum* SCC3193	Clan MA, Family M10	Not shown, but protease has Zn^2+^ and Ca^2+^ binding motifs	EDTA	Unknown	Unknown	In vitro induction by celery and potato extract with a maximum in the early exponential phase and in the presence of PGA at the beginning of stationary phase; in vivo induction in planta in the early stage of infection	The PrtW mutants infected tobacco plants less efficiently in the in vitro culture and showed reduced maceration of potato tubers.	Activity against casein	Synthesis regulated by ExpI and ExpA–ExpS	[28,29]
PrtA from *D. chrysanthemii* B374	Clan MA, Family M10	Not shown but protease has Zn^2+^ and Ca^2+^ binding motifs	EDTA	The N-terminal propeptide is cleaved after secretion	Rich medium during exponential and stationary phase	Unknown	Unknown	Activity against casein	Secretion via T1SS and secretion signal at the C-terminus	[30,31]
PrtB from *D. chrysanthemii* B374	Clan MA and Family M10	Zn^2+^ is required for activity and Ca^2+^ is required for stability	EDTA and phenan-throline	The N-terminal propeptide is cleaved after secretion in the rich medium (not minimal)	Rich medium during exponential and stationary phase	Unknown	Unknown	Activity against casein	Secretion via T1SS and secretion signal at the C-terminus	[30,32]
PrtC from *D. chrysanthemii* B374	Clan MA and Family M10	Zn^2+^ is required for activity, and Ca^2+^ and Mg^2+^ for stability	EDTA and phenan-throline	The N-terminal propeptide is cleaved after secretion in the rich medium (not minimal)	Rich medium during exponential and stationary phase	Unknown	Unknown	Activity against casein	Secretion via T1SS and secretion signal at the c-terminus	[30,32]
PrtG from *D. chrysanthemii* B374	Clan MA, Family M10	Unknown	EDTA	The N-terminal propeptide is cleaved after secretion	Rich medium	Unknown	Unknown	Activity against gelatin	Secretion via T1SS and secretion signal at the C-terminus; low abundance protease	[33]
Prt2 form *X. campestris* pv. *campestris*	Unassigned	Zn^2+^ is required for activity, in addition, Ca^2+^, Mn^2+^, and Mg^2+^ are required for activity and/or stability	EDTA; phenan-throline	Unknown	Rich medium and minimal medium supplemented with plant cell walls	Unknown	The mutant lacking Prt1 (serine protease) and Prt2 showed reduced maceration symptoms in the turnip leaves	Optimal pH of around 8; activity against casein	Prt2 and Prt1 are major proteases of the vascular pathovars of *X. campestris*	[26]
Prt3 from *X. campestris* pv. r*aphanin* and *X. campestris* pv. *armoraciae*	Unassigned	Zn^2+^	Phenan-throline, DTT, and insensitive to EDTA	Probably by cutting the signal peptide	Secreted in rich medium and *in planta*	Unknown	Unknown	Optimal pH of 8–9; activity against β-casein	The major protease of the mesophilic pathovars of *X. campestris*	[25]

Abbreviatons: PGA—polygalacturonic acid; DTT—ditiotreitol; EDTA—(ethylenedinitrilo)tetraacetic acid; EGTA—ethylene glycol-bis(2-aminoethylether)-N,N,N’,N’-tetraacetic acid, T1SS—type one secretion system, BSA—bovine serum albumin.

## Abbreviations

AAA+ family of ATPases	ATPases associated with diverse cellular activities
AXR	auxin-resistant
BAK1	brassinosteroid insensitive 1-associated kinase 1
BIK1 kinase	botrytis-induced kinase 1
BSA	bovine serum albumin
DFP	diisopropylfluorophosphate
Dps	DNA-binding protein of starved cells
DTT	ditiotreitol
ECF	extracytoplasmic function
EDTA	(ethylenedinitrilo)tetraacetic acid
EF-Tu	elongation factor thermos unstable
EGTA	ethylene glycol-bis(2-aminoethylether)-N,N,N’,N’-tetraacetic acid
EAR	ERF-associated amphiphilic repression
ERF	ethylene response factor
ET	ethylene
ETI	effector-triggered immunity
FLS2	flagellin sensing 2
HopZ4	hypersensitivity and pathogenesis-dependent outer protein Z4
HR	hypersensitive response
*hrc* genes	HR and conserved genes
Hrp	hypersensitive response and pathogenicity
HtrA	high temperature requirement A
JA	jasmonic acid
JAZs	jasmonate zimdomain proteins
MarA	multiple antibiotic resistance activator
MKK	mitogen-activated protein kinase kinases
MPK	mitogen-activated protein kinase
NOI	nitrate-induced
NPR1	Nonexpressor of Pathogenesis-Related1
OEC	oxygen evolving complex
PAMPs	pathogen-associated molecular patterns
PBS1	AvrPphB susceptible1
PDZ	post synaptic density protein 95, Drosophila disc large tumor suppressor, and Zonula occludens-1 protein domain
Pel	pectate lyase
PGA	polygalacturonic acid
PRRs	pattern recognition receptors
PTI	pattern-triggered immunity
PBL1	PBS-like 1
PS II	photosystem II
RPM1	resistance to *Pseudomonas syringae* pv. *maculicola*1
R-protein	resistance (R) protein
RLCK	receptor-like cytoplasmic kinase
RIN4	RPM1-interacting protein 4
ROS	reactive oxygen species
RsmA	a small RNA-binding protein
RpoE	sigma E
Rcs	regulator of capsule synthesis
RpoS	sigma S
RPS5	resistance to *Pseudomonas syringae* 5
RPT6	regulatory particle AAA-ATPase 6
SA	salicylic acid
SDS	sodium dodecyl sulfate
SERK	somatic embryogenesis receptor like kinase
Sox	Sry-related high-mobility-group box
sRNAs	small RNAs
SsrA	peptide tag encoded by small stable RNA A
SspB	stringent starvation protein B
SUMO	small ubiquitin-like modifier
Tsp	tail-specific protease
T1SS	type one secretion system
T3SS	type three secretion system
ULPs	ubiquitin-like protein proteases
UmuD’	umu: UV mutagenesis
wt	wild type
Xop	*Xanthomonas* outer protein
Yop	*Yersinia* outer protein
